# Efficacy of interventions to increase the uptake of chlamydia screening in primary care: a systematic review

**DOI:** 10.1186/1471-2334-11-211

**Published:** 2011-08-05

**Authors:** Rebecca J Guy, Hammad Ali, Bette Liu, Simone Poznanski, James Ward, Basil Donovan, John Kaldor, Jane Hocking

**Affiliations:** 1 The Kirby Institute, Sydney, New South Wales, Australia; 2 Centre for Women's Health, Gender and Society, Melbourne School of Population Health, University of Melbourne, Melbourne, Victoria, Australia; 3 Sydney Sexual Health Centre, Sydney Hospital, Sydney, New South Wales, Australia

## Abstract

**Background:**

As most genital chlamydia infections are asymptomatic, screening is the main way to detect and cases for treatment. We undertook a systematic review of studies assessing the efficacy of interventions for increasing the uptake of chlamydia screening in primary care.

**Methods:**

We reviewed studies which compared chlamydia screening in the presence and the absence of an intervention. The primary endpoints were screening rate or total tests.

**Results:**

We identified 16 intervention strategies; 11 were randomised controlled trials and five observational studies, 10 targeted females only, five both males and females, and one males only. Of the 15 interventions among females, six were associated with significant increases in screening rates at the 0.05 level including a multifaceted quality improvement program that involved provision of a urine jar to patients at registration (44% in intervention clinics vs. 16% in the control clinic); linking screening to routine Pap smears (6.9% vs. 4.5%), computer alerts for doctors (12.2% vs. 10.6%); education workshops for clinic staff; internet-based continuing medical education (15.5% vs. 12.4%); and free sexual health consultations (16.8% vs. 13.2%). Of the six interventions targeting males, two found significant increases including the multifaceted quality improvement program in which urine jars were provided to patients at registration (45% vs. 15%); and the offering by doctors of a test to all presenting young male clients, prior to consultation (29 vs. 4%).

**Conclusions:**

Interventions that promoted the universal offer of a chlamydia test in young people had the greatest impact on increasing screening in primary care.

## Background

Infection with *Chlamydia trachomatis *is a significant public health problem. In women it causes adverse health consequences such as pelvic inflammatory disease which in turn can lead to tubal factor infertility and ectopic pregnancy[1,2]. As over 80% of infections are asymptomatic, screening on the basis of epidemiological risk factors such as age and sexual history is the main way to detect cases[1,2]. Clinical guidelines recommend chlamydia screening in all sexually active young females in many countries[3-5], and to young males in some countries[4].

Primary care clinics play an important role in the prevention and management of sexually transmissible infections (STIs). A large proportion of young people attend primary care clinics each year for one reason or another[6,7], and most chlamydial infections are diagnosed in this setting[6,8]. However, despite the central role of primary care in chlamydia management, the proportion of sexually active young people attending these clinics who are offered screening at the time of their visit is low in many countries ranging from 3.3% of 15-24 year females and 1.0% of males in the South East of England in 2006/07[9]: and 12.5% of young sexually active females and 3.7% of males in Australia in 2008[7].

A systematic review in 2006 by Ginige et al identified four published trials of interventions to increase chlamydia screening in primary care and found that educational packages targeting primary care physicians, and the elimination of barriers to screening within clinic systems were effective at increasing screening[10]. Since then, a number of new publications have reported on the evaluation of interventions to increase chlamydia screening in primary care clinics among patients attending for routine consultations. This systematic review aimed to provide an updated synthesis of studies examining the efficacy of these interventions, including the trials considered in the 2006 review.

## Methods

The systematic review was conducted according to the PRISMA statement[11].

### Review strategy

A publication was considered for inclusion if it reported on the evaluation of an intervention to increase chlamydia screening rates in a primary care clinic, through a comparison with chlamydia screening rates (proportion of patients screened within a given time period) in a control group or control time period. A primary care clinic was defined as a health service that provides the first point of entry into the health care system, addresses the vast majority of patient concerns and needs, and is the ongoing focal point for all of a patient's health care requirements. This definition excluded more specialised services, such as sexual health clinics, family planning clinics, and pharmacies.

Following Ginige et al (2006), who searched the Medline database for studies prior to 2005 using the words chlamydia, screening, intervention, primary care and GPs, we used the search terms as listed below, and extended the search to additional electronic databases (Medline, PubMed, EMBASE, the Cochrane Controlled Trials Register and the Australian New Zealand Clinical Trial Registry), to the end of September 2010. Only English language publications were included. Reference lists of selected studies were also checked for other potentially relevant studies.

1. Chlamydia infections, or Chlamydia, or Chlamydia trachomatis, AND

2. Testing or screening, AND

3. Intervention, or trial, or intervention studies AND

4. General practice or general practitioner or GP or primary care

The papers were reviewed and information extracted by two authors independently. Disagreements were resolved by discussion and consensus.

Publications were excluded that did not incorporate a control group; reported on screening rates in the absence of a specific intervention; described chlamydia or STI screening programs in clinic or community settings other than primary care; described surveys of patients or providers about chlamydia screening; or did not report original data.

For each paper that met the inclusion criteria, information was extracted on the clinic location, the target population, the intervention strategy, the study design, the sample size, the statistical tests used and the outcomes of the evaluation including chlamydia screening rates or number of tests.

### Analysis

We conducted a frequency analysis of information related to the clinic (location, type), intervention type and evaluation methods (sample size, design, analytical techniques, time period of the evaluation and reported outcomes).

The primary outcome for each study was the screening rate, defined as the proportion of patients attending the clinic who were screened for chlamydia. For studies that did not report this proportion, we accepted the total number of tests done as an alternative.

From each study, we abstracted the odds ratio (OR) or relative risk (RR) indicating the proportion tested in the intervention group compared to controls. For studies which did not report a measure of this kind we calculated the outcomes using Stata statistical software[12], including 95% confidence intervals, if the necessary data were provided in the paper.

## Results

### Search outcomes

**Figure 1:**
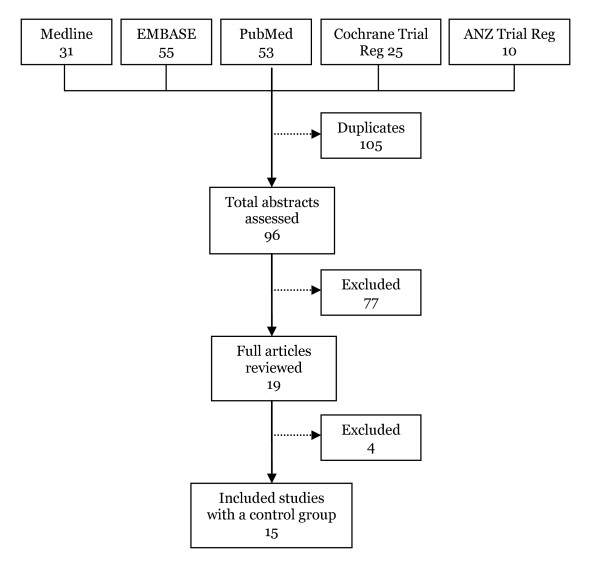
**Search results**. ANZ = Australia New Zealand

Using the search words 'chlamydia' and 'screening', 'intervention' and 'general practice', and variations of these terms, 96 articles were identified and the abstracts from these articles were reviewed (Figure 1). A total of 81 papers were excluded because they either described interventions to improve outcomes other than chlamydia screening (n = 25); described chlamydia or STI screening programs in clinic or community settings other than primary care (n = 15); were reviews or commentaries which did not contain original data (n = 13); described surveys of patients or providers about chlamydia screening (n = 5); described a cross sectional or cohort study which reported STI incidence or prevalence, screening rates or risk factors (n = 4); described mathematical transmission models or cost-effectiveness analyses of the impact of chlamydia screening (n = 4); described a study of non-genital chlamydia (n = 5); described a chlamydia immunological study (n = 1); was a case report (n = 1); there was no control group (n = 2); the paper did not contain any data (n = 6).

### Overview of papers included

The remaining 15 papers [6,13-26] were included in the review; four had been the subject of an earlier review by Ginige and colleagues[13,23-25]. Studies were conducted in Australia (n = 5), the US (n = 5), the UK, Scotland, Belgium, Denmark and New Zealand (NZ) (1 each). Two papers[19,22] each reported on the evaluation of two distinct intervention strategies, and two papers reported on the evaluation of the same intervention strategies (one in females, one in males)[23,24] giving a total of 16 strategies evaluated across the 15 papers. Of the 16 intervention strategies; 10 targeted females only, five both males and females, and one males only.

As shown in Table 1, we grouped the 16 strategies to increase chlamydia screening into six broad categories, based on the methodological descriptions provided in the reports: medical record prompts[19,22,26]; clinician incentives[15,21]; alternative specimen collection approaches[6,17]; clinician education[13,14,25]; patient education[16]; and quality improvement programs[20,22-24].

**Table 1:** Studies of interventions to increase screening in females (n = 15)

Author surname, year	Country	Intervention type	Evaluation design	Clinics (n)	Target age group (yrs)	Intervention phase	Intervention group		Control group		Statistical findings reported**	Crude RR (and 95% CI) calculated by reviewer**
									
							Patients (n)	% screened	Patients (n)	% screened		
Walker[26] 2010	Aust		RCT	66	16-24	During	12098	12.2%	12924	10.6%	OR = 1.3 (95%CI:1.1-1.4)*^A^*	
								
		Prompt				Before	11518	8.3%	11704	8.8%		
		
Scholes[22] 2006	US		RCT	23	14-20	During	1777	42.6%	1732	40.8	OR = 1.0 (95%CI:0.9-1.2)^B^	
		
McNulty[19] 2008	UK		RCT	44	16-24	During	-*	-*	-*	-*	RR = 1.0 (95%CI:0.8-1.2)^C^	

Bilardi[15] 2010	Aust		RCT	12	16-24	During	1589	13.4%	1792	8.8%	OR = 0.9 (95%CI:0.6-1.2)^D^	
								
		Incentive				Before	2662	11.5%	2689	6.2%		
		
						During	4018	16.8%	9068	13.2%		
								
Morgan[21] 2009	NZ		Non-RCT	49	16-24	Roll out	5368	15.5%	12124	13.7%	NR	1.3 (1.2-1.4)^E^
								
						Before	2676	13.9%	6077	13.0%		

Bowden[17] 2008	Aust	Alternative specimen collection	RCT	31	16-25	During	16082	6.9%	10794	4.5%	OR = 2.1 (95%CI:1.3-3.4)^F^	

Verhoeven[25] 2005	Belgium		RCT	36	< 35 yr	During	-*	7#	-*	4.72#	p = 0.106^G^	1.5 ^E, H^
		
Burstein[18] 2005	US		Non-RCT	NS	15-26	During	-*	32%	-*	-*	NS	1.1^H^
								
		Doctor education				Before	-*	30%	-*	-*		
		
Armstrong[14] 2003	Scotland		Non-RCT	2	15-24	During	-*	146^##^	-*	138^##^	NR	1.1^E, H^
								
						Before	-*	53^##^	-*	113^##^		
		
Allison[13] 2005	US					After	-*	15.5%	-*	12.4%		
								
			RCT	191	16-26	During	-*	13.3%	-*	13.0%	p = 0.04^I, J^	1.3^H, J^
								
						Before	-*	16.2%	-*	18.9%		
		
McNulty[19] 2008	UK		RCT	82	16-24	During	-*	-*	-*	-*	RR = 1.3 (95%CI:1.1-1.6)^C^	

Bilardi[16] 2009	Aust	Patient education	Non-RCT	3	16-24	During	2002	6.4%	-*	-*	p = 0.95^G^	1.0 (0.8-1.2)
								
						Before	1548	6.3%	-*	-*		

Schafer[23] 2002	US		RCT	10	14-18	During	1092	43.8%	1299	15.6%	p < 0.01	2.8 (2.4-3.2)^E^
								
		Quality improvement program				Before	80	5.0%	86	14.0%		
		
Scholes[22] 2006	US		RCT	23	14-25	During	5650	42%	6105	40.1%	OR = 1.0 (95%CI:0.9-1.1)^B^	
		
Merritt[20] 2007	Australia		RCT	6	15-24	Late-intervention	-*	10.2%^	-*	-*	NR	1.5^H, I^
								
						Before	-*	6.7%^	-*	-*		

**Higher odds ratio or relative risk means intervention leads to greater screening.

Aust-Australia, US = United States, UK = United Kingdom, NZ = New Zealand, RCT = Randomised controlled trial, OR-odds ratio, RR-relative risk, M = male F = female NR = not reported, NS = not significant. *Information not reported, #mean tests per GP, ##total tests, ^available

for four of six practices

A = Author conducted a mixed effect logistic regression with a 3-level hierarchy (patients, individual GPs and GP clinics)

B = Author conducted chi-square tests and logistic regression model

C = Author conducted a multi-level model with aggregate baseline tests and positivity included as co-variates. Unit of analysis was GP practice

D = Author conducted a mixed effect logistic regression with a 2-level hierarchy (patients, individual GPs)

E = Screening rate in intervention clinic compared to control clinic during intervention period only

F = Author conducted logistic regression adjusted for clustering within general practices = number of female doctors per practices and number of doctors enrolled in the practice were included in the model

G = Author conducted a test for equality in proportions

H = Insufficient information to calculate 95% CI

I = Author conducted an intention to treat analysis at the clinic level comparing mean post-intervention screening rates for the two groups. A general linear model adjusted for pre intervention done and intra intervention screening rates using repeated-measures analysis

J = Screening rate in intervention clinic compared to control clinic post intervention

Of the 16 intervention strategies, eleven were described as having been evaluated using a randomized controlled trial (RCT) design[13,15,17,19,22-24,26] while five reported using an observational design with control clinics or a control period[6,14,16,20,21,25], One of the randomized designs only involved random assignment between two clinical sites. Because this very limited degree of randomization can not overcome the potential for major confounding due to differences between sites that are unrelated to the intervention, we reclassified it for the purpose of the review as observational[14] Of the other five observational evaluations, two involved non-random allocation of clinics to the intervention, with comparison being made to control clinics and to the pre-intervention period in the intervention clinics [6,21], and the other three used a before and after design within the same group of clinics[16,20,25].

The primary study outcomes reported were clinic screening rates (13 studies)[6,13,15-17,19,21-24,26], total tests done (two studies)[14,20], and mean number of tests per clinician (one study)[25]. One of the studies that reported total tests also described the screening rate in four of six participating clinics[20]. In one study the primary outcome data was screening rates at the clinic level but only one doctor in each clinic participated in the intervention [13]. In the RCTs, the total number of participating clinics ranged from 12-191. In the observational studies, the range was 2-49. Three evaluations did not report statistical tests required to determine if screening rates between intervention and control groups were significantly different [14,20,21]. None of the observational studies reported any form of adjustment in their analyses for differences in baseline characteristics, including chlamydia screening rates, between the intervention and control clinics, although there were such differences. For example in the NZ study, clients of intervention clinics were more likely to be of a lower socioeconomic status, Māori and rural population [21].

### Impact of interventions among females

**Figure 2:**
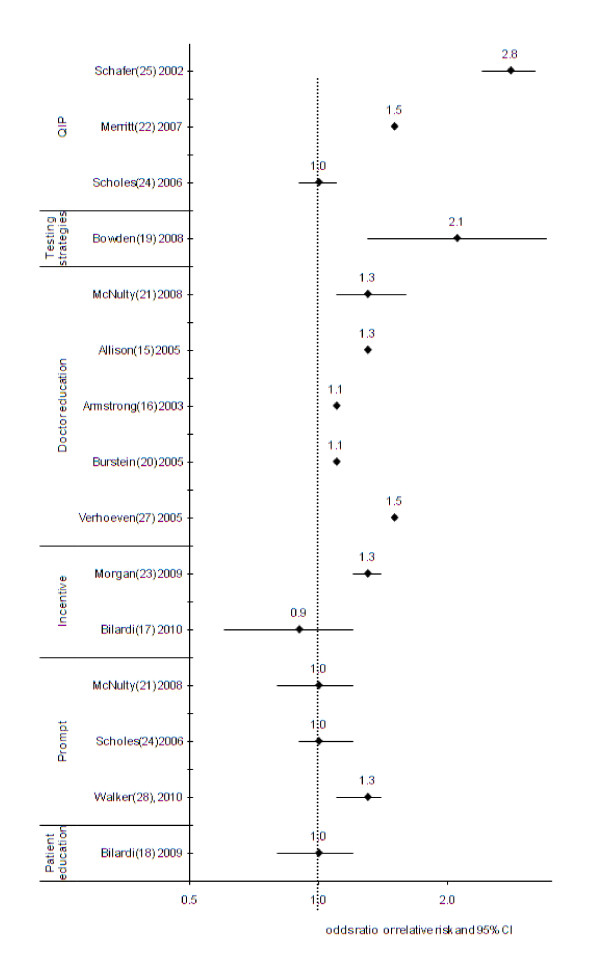
Odds ratio or relative risk* of intervention studies to increase chlamydia screening in females, by intervention strategy type (n = 15). CI = Confidence interval, QIP = quality improvement program *Higher odds ratio or relative risk means intervention leads to greater screenin

There were 15 intervention strategies which targeted females. Six of the 15 intervention strategies were significantly associated with increased chlamydia screening at the 0.05 level[13,17,19,21,23,26] (Table 1, Figure 2).

A large increase was observed in a multifaceted quality improvement program targeting the screening of 14-18 year olds, in whom a screening rate of 65% was achieved in females by the end of the program compared to the 21% in the same time period at control clinics (p < 0.001)[23]. The increase was achieved within a few months, and sustained for 18 months. Clinicians in the intervention group participated in a 4-stage clinical improvement initiative consisting of an baseline analysis of the gap between current and best screening practice in participating clinics, capacity building, developing a clinic flow chart and promotional material, monthly meetings of the team members to identify barriers to screening and strategies to overcome them, development of performance indicators, and introduction of universal urine specimen collection from all adolescents at registration, prior to examination (Table 1, Figure 2).

Other strategies that were associated with significant but smaller increases in screening in females included linking chlamydia screening with a Pap smear in a RCT in Australia[17]. In this study, about 25% of all chlamydia screening in both study groups were conducted among females aged 30-39 years with a very low chlamydia positivity obtained (< 1%). Integration of computer alerts within patient management systems based on age group (16-24) and female sex of clients in Australia, also demonstrated a small increase in screening [26]. An evaluation of the impact of provision of funding for free general practice sexual health visits for registered adolescents under-25 year in New Zealand reported a 16.8% screening rate among females attending the intervention clinics, compared to 13.2% in the control clinics with no other statistical tests reported[21]. Small but significant increases in chlamydia screening were also reported due to an interactive educational workshop for clinic staff promoting screening in 16-24 year old females in the UK[19] and an internet-based education program for doctors, promoting screening in 16-24 year old females (Table 1, Figure 2)[13].

Two intervention studies reported increases in screening but did not include any statistical analysis to demonstrate the increases were significantly different from control groups[14,20], One was a multi-faceted quality improvement program in Australia that introduced chlamydia screening during practice visits for other purposes. Doctors were trained to developed tactics for introducing the chlamydia test and had regular meetings to discuss progress. However, the increase was not uniform or sustained[20]. The other study was conducted in Scotland and introduced an external advisor at one clinic to raise awareness of chlamydia and train staff on guidelines, and compared chlamydia screening to another clinic without an advisor[14]. The number of chlamydia tests performed in females increased by 176% during the 6 month period when the advisor was present compared to a six month period before, with 70% of tests conducted by practice nurses. In the control clinics screening only increased by 22% (Table 1, Figure 2)[14].

The remaining eight intervention strategies that did not result in an increase in chlamydia screening were: a written chart prompt in the US[22]; an educational package (video and text) on communication skills for sexual history taking in Belgium[25]; clinician referral of patients to an interactive website called "Youth Check Your Risk' post-consultation in Australia[16]; laboratory forms modified to include information about chlamydia screening in the UK[19]; screening recommendations and provider training in the US[18]; a $5 (AUD) incentive in Australia[15]; and a multifaceted quality improvement program in the US[22]. The quality improvement program included selecting 'leaders' within each clinic, an initial training session, regular feedback on screening performance, provision of guidelines, a prompt with Pap test, posters and chlamydia information. The control group received the standard chlamydia screening guideline - this was placed on each clinic's intranet and all physicians were advised of its posting. The intervention did not significantly affect overall screening rates, but did lead to a significant increase in screening in women having a Pap test (74.6% versus 70.4%, p = 0.04) and a significant increase in screening in women undergoing a physical examination (64.4% versus 59.7%, p < 0.01) (Table 1, Figure 2).

### Impact of interventions among males

**Figure 3:**
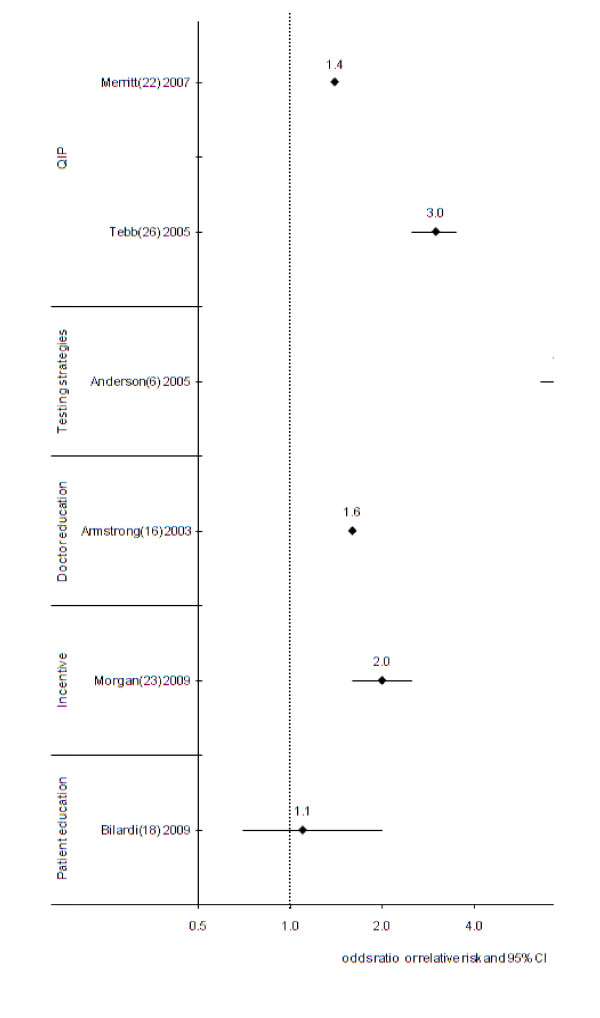
Odds ratio or relative risk* of intervention studies to increase chlamydia screening in males, by intervention strategy type (n = 6). CI = Confidence interval, QIP = quality improvement program, *Higher odds ratio or relative risk means intervention leads to greater screening.

Of the six intervention strategies which targeted males, two were significantly associated with increased chlamydia screening[6,24]. The greatest impact (673% increase) in chlamydia screening was observed in a Danish study in which doctors were asked to test all 16-25 year old males whom they saw for any reason, by use of a first-catch urine sample. Control clinics comprised all other clinics in Denmark. Baseline screening in control and intervention clinics was 3.4% and 3.7% respectively and over a 12 month period following the intervention, the study found screening rates were 29% in the intervention clinics compared with 4% in the control clinics (p < 0.001)[6]. The multifaceted quality improvement which achieved high screening rates in females[24], also led to a large increase in 14-18 year old males, in whom uptake of 49% was achieved by the end of the program compared to the 5% in the same time period at control clinics (p < 0.001)[24]. The increase was achieved within a few months, and sustained for at least 18 months (Table 2, Figure 3).

**Table 2:** Studies of interventions to increase screening in males (n = 6)

Author surname, year	Country	Intervention type	Evaluation design	Clinics (n)	Target age group (yrs)	Intervention phase	Intervention group		Control group		Statistical findings reported**	Crude RR (and 95% CI) calculated by reviewer**
									
							Patients (n)	% screened	Patients (n)	% screened		
						During	4190	4.2%	8524	2.1%		
								
Morgan[21] 2009	NZ	Incentive	Non-RCT	49	16-24	Roll out	5588	3.4%	11333	2.1%	NR	2.0 (1.6-2.5)^A^
								
						Before	2833	3.0%	5529	1.7%		

Anderson[6] 2005	Denmark	Alternative specimen collection	Non-RCT	3	16-25	During	617	29.4%	11204	3.8%	p < 0.01^B^	7.7 (6.6-9.0)^A^
								
						Before	607	3.7%	12007	3.4%		

Armstrong[14] 2003	Scotland	Doctor education	Non-RCT	2	15-24	During	-*	16^	-*	10^	NR	1.6^C, D^
								
						Before	-*	4^	-*	8^		

Bilardi[16] 2009	Aust	Patient education	Non-RCT	3	16-24	During	965	3.0%	-*	-*	p = 0.77^B^	1.1 (0.7-2.0)
								
						Before	732	2.7%	-*	-*		

Tebb[24] 2005	US		RCT	10	14-18	During	990	44.9%	1024	15.1%	p < 0.01	3.0 (2.5-3.5)
								
		Quality improvement program				Before	76	2.6%	61	7.0%		
		
Merritt[20] 2007	Australia		RCT	6	15-24	Late-intervention	-*	6.3%#	-*	-*	NR	1.4^C^
								
						Before	-*	4.5%#	-*	-*		

** Higher odds ratio or relative risk means intervention leads to greater screening

Aust-Australia, US = United States, UK = United Kingdom, NZ = New Zealand, RCT = Randomised controlled trial, OR-odds ratio, RR-relative risk, M = male F = female NR = not reported,*Information not reported, ^total tests # available for four of six practices

A = Screening rate in intervention clinic compared to control clinic during intervention period only

B = Author conducted a test for equality in proportions

C = Insufficient information to reviewers to calculate 95% CI

D = Reviewers compared total tests in intervention clinic to control clinic during intervention period only

Two intervention studies reported increases in screening in males but did not include any statistical analysis to demonstrate whether the increases were significantly different from control groups[14,20]. One was the multi-faceted quality improvement program in Australia that introduced chlamydia screening during practice visits for other purposes[20]. The second study was conducted in Scotland and introduced an external advisor at one clinic to raise awareness of chlamydia and train staff on guidelines, and compared another clinic where there was no advisor[14]. The number of chlamydia tests performed in males in a six month period before the intervention was 4, increasing to 16 during the 6 month period when the advisor was present, compared with the control clinic clinics where 8 tests were conducted before the intervention, and 10 when the advisor was present (Table 2, Figure 3).

The remaining two intervention strategies that did not result in an increase in chlamydia screening were: screening recommendations and provider training in the US[18]; and provision of funding for free general practice sexual health visits for registered adolescents under-25 year in New Zealand[21] (Table 2, Figure 3).

### Interventions which targeted males and females

Five studies targeted both males and females [14,16,20,21,23,24], and four of these found a greater increase in screening in males, compared with females (Table 2, Figure 3)[14,16,21,23,24], while one which used the strategy of linking screening with women's health-related consultations demonstrated a greater increase in screening in females (Table 3) [20].

**Table 3:** Studies of interventions to increase screening in both sexes, by sex (n = 5)

Author surname, year	Intervention type	Sex	Intervention phase	Intervention group		Control group		Statistical findings reported**	Crude RR (95% CI) calculated by reviewer**
						
				Patients (n)	% screened	Patients (n)	% screened		
			During	4018	16.8%	9068	13.2%		
					
		F	Roll out	5368	15.5%	12124	13.7%	NR	1.3 (1.2-1.4)
					
Morgan[21] 2009	Incentive		Before	2676	13.9%	6077	13.0%		
		
			During	4190	4.2%	8524	2.1%		
					
		M	Roll out	5588	3.4%	11333	2.1%	p = 0.05^A^	2.0 (1.6-2.5)
					
			Before	2833	3.0%	5529	1.7%		

Merritt[20] 2007	Quality improvement program	F	Late-intervention	-*	10.2%^C^	-*	-*	NR	1.5^B^
					
			Before	-*	6.7%^C^	-*	-*		
		
		M	Late-intervention	-*	6.3%^C^	-*	-*	NR	1.4^B^
					
			Before	-*	4.5%^C^	-*	-*		

		F	During	-*	146^	-*	138^	NR	1.1^B, D^
					
Armstrong[14] 2010	Doctor education		Before	-*	53^	-*	113^		
		
		M	During	-*	16^	-*	10^	NR	1.6^B, D^
					
			Before	-*	4^	-*	8^		

Bilardi[16] 2009	Patient education	F	During	2002	6.4%	-*	-*	1.0^E^	1.0 (0.8-1.2)
					
			Before	1548	6.3%	-*	-*		
		
		M	During	995	3.0%	-*	-*	0.8^E^	1.1 (0.7-2.0)
					
			Before	752	2.7%	-*	-*		

Schafer[23] 2002,	Quality improvement program	F	During	1092	43.8%	1299	15.6%	p < 0.01^F^	2.8 (2.4-3.2)
					
Tebb[24] 2005			Before	80	5.0%	86	14.0%		
		
		M	During	990	44.9%	1024	15.1%	p < 0.01^G^	3.0 (2.5-3.5)
					
			Before	76	2.6%	61	7.0%		

** Higher odds ratio or relative risk means intervention leads to greater screening

OR-odds ratio, RR-relative risk, * Information not reported, M = male F = female, NR = not reported

^Total tests

A = Authors conducted a t-test for differences in the proportion of tests conducted in males and 16-24 year olds in the intervention practices compared to control practices

B = Insufficient information to reviewers to calculate 95% CI

C = Screening rates based on 4 of the six clinics in the intervention clinics only

D = Reviewers compared total tests in intervention clinic to control clinic during intervention period only

E = Author conducted a test for equality in proportions

F = Authors assessed the statistical significance of the time by group effect using an F test

G = Authors adjusted for differences in ethnicity between study groups at baseline

## Discussion

In this review, we found that a variety of new approaches are being evaluated for their potential to increase uptake of chlamydia screening among young people attending primary care clinics. A range of potentially effective strategies were identified in these studies, with six out of the 15 interventions targeting young females and two of the six interventions among young males finding statistically significant increases in screening rates.

The two studies with greatest effect from the US and Denmark, involved systems change to enable all patients to be offered a chlamydia test. In contrast, the effect of the multi-faceted quality improvement programs by Scholes[22] and Merritt[20] which found no or limited increases in screening rates, may have been constrained because the interventions didn't cause sufficient systems change to results in the universal offer of screening, instead it was more opportunistic screening often linked with Pap smears. However in the study by Scholes[22] the lack of effect may also have been because the screening rates in the control population were already relatively high (40.1%). This study placed standard chlamydia screening guidelines on each clinic's intranet in the intervention and control clinics, which in turn alerted all doctors of the need for screening.

The main limitation of linking chlamydia screening with Pap smears is that it is unlikely to capture young women (< 20 years) who are ineligible for Pap smears in most developed countries, as demonstrated the study by Armstrong[14], where most of the increased screening occurred outside the target age range, and Bowden where 25% of chlamydia screening in both study groups were conducted among women aged 30-39 years[17]. Furthermore the extent of chlamydia screening would be reliant on recommended Pap smear screening intervals and age groups which do not necessarily coincide with that recommended for chlamydia screening. The strength of this strategy is that screening could be conducted by practice nurses during women's health consultations, overcoming the barriers of insufficient time raised by clinicians[20,27-29], and concerns that discussing chlamydia in a consultation unrelated to sexual health might upset patients[20,27,28,30].

Two doctor education strategies resulted in small but statistically significant increases in screening; interactive education workshops in general practice clinics in the UK[19], and internet-based Continuing Medical Education (CME), involving 4 modules released every 3 months to primary care physician offices in the US[13]. The interactive workshop is likely to require significant staff resources to roll out at a national level. The CME strategy may be cheaper but would be unlikely to reach all clinicians, with CME activities generally taken up by those clinicians interested in the area of sexual health.

Computer alerts were also shown in one study in Australia to achieve a small improvement in provider behaviour[26]. These findings are consistent with a Cochrane review on the effects of on-screen, point of care computer reminders on processes and outcomes of care, which found that computer reminders achieved a median improvement in process adherence of 3.8% (IQR: 0.4% to 16.3%) for test ordering[31]. In contrast, more passive prompts such as attaching a reminder sticker to medical records[19], and including chlamydia information on laboratory result forms[22], did not significantly increased chlamydia screening.

Although Bilardi demonstrated that a small incentive paid to practitioners did not increase chlamydia screening rates in Australia[15], the study was limited by the fact that clinicians did not receive the payment until the conclusion of the trial and there was limited contact and ongoing communication from study investigators during the trial.(Personal communication - Hocking) The authors recommended future studies should include a higher incentive, and/or be associated with more regular feedback[15]. Incentive payments were used in the chlamydia screening pilots conducted in the UK. General practitioners were reimbursed up to £20 per eligible person screened, with screening acceptance rates within clinics of up to 81%[32]. However, once the chlamydia screening program was rolled out across the country, incentive payments were removed and screening participation rates within clinics fell to below 10%[33], Incentive payments were offered to general practitioners to enrol patients for chlamydia screening in Amsterdam with 94% acceptance[34]. However, without RCT evidence, it is not possible to predict how well general practitioners would respond to an incentive payment to increase chlamydia screening rates.

None of the studies specifically explored the role practice nurses or other clinic staff could play in chlamydia screening in primary care, although in the study by Armstrong et al[14] practice nurses conducted 70% of screening by linking it with Pap smears, and in the study by Shafer[23] and Tebb[24] the urine jar was given to patients by reception staff at registration, prior to the examination. It is possible that practice nurses, and other generalist accredited health workers could play a greater role in chlamydia screening in primary care clinics.

This review has some methodological limitations. First, we did not search the grey literature so it is possible that some evaluations were not identified, particularly those with a negative outcome. Second, it is possible that in the observational studies as the intervention was not randomised any imbalances in factors that may have influenced chlamydia screening rates may have biased the study findings. Third, the populations and health care systems in the study settings also varied, so the extent to which the findings would apply to other settings is uncertain. For example, the multi-faceted quality improvement intervention reported by Shafer[23] and Tebb[24] was conducted in paediatric clinics whereas as a number of other studies were in general practice clinics. Fourth, due to the heterogeneity of the interventions and outcomes we were unable to pool the outcomes to determine a summary effect.

To maximise the value of future evaluations, attention should be paid to methodological issues, including conducting statistical tests for significance, taking into account the pre-intervention screening rates or differences in other baseline characteristics into the analysis, and reporting screening rates rather that total tests done. The lack of reporting of screening rate in some studies is likely to be related to the need to obtain the number of unique patients from general practice patient management systems, which can now be facilitated through data extraction software[35].

The question remains as to which of these strategies should be employed in primary care to increase chlamydia screening. It is not clear which would be the most cost-effective due to the absence of costing data for all the strategies to enable comparison of impact per unit cost. While the effect of the intervention seen in the study by Shafer[23] and Tebb[24] appeared to be relatively large, the intensity and complexity of the strategies employed may be too difficult to implement universally. By contrast, the computer alert evaluated by Walker and colleagues[26] would be easier to disseminate with little impact on a general practitioner's time but would not achieve coverage levels of sufficient magnitude to have an impact on population prevalence as demonstrated in mathematical modelling[36]. Sustainability of the interventions on chlamydia screening is another issue which was not specifically addressed by most studies and this factor should also be considered in selecting appropriate strategies.

## Conclusion

Despite the limitations to the studies published, it appears that interventions that provide easy and systematic means of offering a chlamydia test to all eligible clients had the greatest impact on increasing screening in primary care. Our review focused on studies of young people already attending primary care settings. A fundamental determinant of the success of any clinic-based strategy is the extent to which young people can access clinical services for their sexual health needs. Therefore in applying our findings to population level health advocacy, it is necessary to consider the level of access to primary care clinics in that setting, and the potential to supplement existing clinical services with strategies such as testing programs in non-clinical settings[37].

## Competing interests

The authors declare that they have no competing interests.

## Authors' contributions

RG conceived the study, coordinated the study, extracted the data and drafted the manuscript. HA conducted the literature search and checked the data in collaboration with RG. JK, JH and BL provided epidemiological advice. JW provided advice on the interpretation of the results. All authors read and approved the final manuscript.

## Pre-publication history

The pre-publication history for this paper can be accessed here:

http://www.biomedcentral.com/1471-2334/11/211/prepub
